# Deep Learning-Based Eye-Writing Recognition with Improved Preprocessing and Data Augmentation Techniques

**DOI:** 10.3390/s25206325

**Published:** 2025-10-13

**Authors:** Kota Suzuki, Abu Saleh Musa Miah, Jungpil Shin

**Affiliations:** School of Computer Science and Engineering, The University of Aizu, Aizuwakamatsu 965-8580, Fukushima, Japanmusa@u-aizu.ac.jp (A.S.M.M.)

**Keywords:** computer vision, deep learning, eye-tracking, eye-writing, character recognition, signal normalization, Discrete Fourier Transform (DFT), Convolutional Neural Network (CNN), Temporal Convolutional Network (TCN), leave-one-subject-out cross-validation

## Abstract

Eye-tracking technology enables communication for individuals with muscle control difficulties, making it a valuable assistive tool. Traditional systems rely on electrooculography (EOG) or infrared devices, which are accurate but costly and invasive. While vision-based systems offer a more accessible alternative, they have not been extensively explored for eye-writing recognition. Additionally, the natural instability of eye movements and variations in writing styles result in inconsistent signal lengths, which reduces recognition accuracy and limits the practical use of eye-writing systems. To address these challenges, we propose a novel vision-based eye-writing recognition approach that utilizes a webcam-captured dataset. A key contribution of our approach is the introduction of a Discrete Fourier Transform (DFT)-based length normalization method that standardizes the length of each eye-writing sample while preserving essential spectral characteristics. This ensures uniformity in input lengths and improves both efficiency and robustness. Moreover, we integrate a hybrid deep learning model that combines 1D Convolutional Neural Networks (CNN) and Temporal Convolutional Networks (TCN) to jointly capture spatial and temporal features of eye-writing. To further improve model robustness, we incorporate data augmentation and initial-point normalization techniques. The proposed system was evaluated using our new webcam-captured Arabic numbers dataset and two existing benchmark datasets, with leave-one-subject-out (LOSO) cross-validation. The model achieved accuracies of 97.68% on the new dataset, 94.48% on the Japanese Katakana dataset, and 98.70% on the EOG-captured Arabic numbers dataset—outperforming existing systems. This work provides an efficient eye-writing recognition system, featuring robust preprocessing techniques, a hybrid deep learning model, and a new webcam-captured dataset.

## 1. Introduction

Eye-tracking technology, which measures eye movements and gaze direction, has become an essential tool in enabling human–computer interaction (HCI). This technology allows individuals to control devices using their eye movements, providing an intuitive and non-invasive method of interaction. Eye-tracking has been particularly beneficial in assistive technologies, where it is used to support individuals with severe physical impairments, such as those caused by amyotrophic lateral sclerosis (ALS). However, despite its potential, there are significant challenges that hinder the widespread use and accessibility of eye-tracking technology. These challenges include issues with signal accuracy, user variability, and in many systems, reliance on specialized hardware equipment.

### 1.1. Background

Eye-tracking technology has evolved over the years, with significant contributions in the fields of human–computer interaction (HCI), assistive technology, and extended reality (XR) [1,2]. In particular, eye-writing, an alternative communication method that uses eye movements to represent characters, has shown promise for individuals with conditions such as ALS, where traditional communication methods are severely limited. In eye-writing systems, users trace predefined patterns using only their eyes, allowing them to spell out words and sentences. Nevertheless, current systems rely heavily on electrooculography (EOG) sensors, which, although accurate, require complex, specialized hardware setups.

### 1.2. Motivation

The motivation for this research lies in the need to overcome the existing limitations of eye-tracking technology, particularly in terms of accessibility and portability. While EOG-based systems are effective, they are not ideal for everyday use due to their bulky and intrusive nature. On the other hand, vision-based eye-tracking offers the potential to use more accessible and portable equipment, such as standard webcams, which could be integrated into consumer devices like smartphones and laptops. The goal of this study is to develop a more portable and efficient eye-writing recognition system that can be widely used in various contexts, particularly for individuals with severe disabilities.

### 1.3. Existing Work

Eye-writing recognition has been explored by several researchers who have developed various models to improve accuracy and usability. For example, Tsai et al. introduced the concept of eye-writing along with patterns and a heuristic algorithm for recognition [3]. Their proposed symbols for eye-writing consisted of Arabic numerals and arithmetic symbols. In the recognition task, the symbol was estimated by the number of times the symbol trace was turned. Eleven participants were involved in the experiment, and each participant performed five trials. As a result, an accuracy of 75.5% was achieved in recognizing Arabic numerals and arithmetic symbols. Lee et al. employed dynamic time warping (DTW) for the classification of English alphabets [4]. DTW is a method used to measure the similarity between given and sample signals. The dataset consisted of 26 English alphabets and three special characters. They conducted an experiment with 20 participants and achieved an accuracy of 87.38%. Chang et al. described a machine learning model that combined dynamic positional warping (DPW) and support vector machines (SVM) [5]. They conducted experiments on eye-writing of Arabic numbers with 18 participants, and the writer-independent model showed an accuracy of 95.74%.

Fang and Shinozaki studied the recognition of Japanese Katakana using the hidden Markov model (HMM) and DTW [6]. Their proposed eye gestures consisted of 12 strokes of Japanese Katakana. The dataset was collected from six participants, and each participant performed 10 trials. Their model achieved F1-scores of 77.6% for DTW and 86.5% for HMM. Zou and Zhang utilized the Japanese Katakana database and designed a recognition system based on electrooculography (EOG) signals with the CNN architecture [7]. They reported an accuracy of 92.64% for the recognition of Japanese Katakana strokes. In 2021, Chang et al. developed an EOG-based eye writing recognition model using an ensemble deep neural network with inception modules and an ensemble structure [8]. With its model, the classifier achieved an accuracy of 97.78%. Dai et al. introduced a word and character prediction model using 1D CNN-LSTM architectures [9]. They described a new approach to recognizing a sequence of characters from an eye-written word signal. Their proposed model achieved accuracies of 95% for word recognition and 97% for character recognition, respectively. The Japanese Katakana database was used in the same way as in Zou and Zhang’s work. Bature et al. achieved state-of-the-art performance with an accuracy of 96.20% in a newly created eye-writing dataset, utilizing root translation normalization and a Temporal Convolutional Network (TCN) [10]. They evaluated other datasets, including the HideMyGaze, Complex gaze gesture [11], and Japanese Katakana datasets, and achieved accuracies of 98.81%, 97.76%, and 93.51%, respectively. Table 1 presents a summary of existing work, where many researchers have studied eye-writing recognition methodologies.

### 1.4. Research Gaps and Challenges

Despite advances in eye-writing recognition, several challenges remain, particularly in signal length normalization, recognition accuracy, and user adaptability. Many existing systems rely on EOG sensors, which require specialized hardware such as electrodes and are not easily accessible for general users. While some studies have explored infrared camera-based eye-tracking systems, these also depend on dedicated and often expensive equipment.

To the best of our knowledge, few prior studies have investigated eye-writing recognition using webcam-based eye-tracking, despite its potential as a more accessible alternative. Existing approaches instead rely on other modalities: for example, Chang et al. [8] used an EOG-captured dataset and fixed the sample length for all eye-writing data, but this approach oversimplifies the challenge of addressing varying signal lengths, making it difficult to efficiently handle the random length of individual samples of varying duration within a fixed-length framework. In previous studies, such as Bature et al. [10], eye-tracking data was collected using an infrared-camera-based system; however, the methodology for normalizing signal length was not clearly described. Similarly, Zou and Zhang’s [7] eyeSay system also relied on EOG signals rather than vision-based tracking.

Furthermore, the lack of standardized, publicly available datasets for vision-based eye-writing recognition limits the ability to benchmark and compare different methods. Variations in recording times for eye-written characters, ranging from 1.69 to 23.51 seconds in prior studies, further complicate data alignment, since many models—particularly deep learning architectures such as CNNs and TCNs—require fixed-length input signals to operate effectively.

These gaps highlight the need for accessible, vision-based eye-writing systems, robust signal normalization techniques, and the development of standardized datasets to advance the field.

### 1.5. Proposed Model to Solve the Challenges and Contribution

To address the challenges of signal length normalization and improve performance, this study proposes a novel framework for vision-based eye-writing recognition. The system introduces a length normalization technique that reconstructs signals to a uniform length using the Discrete Fourier Transform (DFT), thereby preserving key characteristics of eye-writing patterns while standardizing input lengths. Unlike traditional EOG-based systems, the proposed method employs a standard webcam, making the technology more practical and accessible.

In addition, the model incorporates advanced preprocessing techniques, including DFT-based normalization and initial-point normalization, which shifts trajectories so that the origin (0,0) serves as the starting point in each channel. These steps enhance the system’s robustness against various artifacts in the input data. The proposed model integrates two deep learning architectures—1D Convolutional Neural Networks (CNN) and Temporal Convolutional Networks (TCN)—to jointly capture spatial and temporal features of eye-writing patterns. This hybrid design enables effective recognition with improved accuracy and usability.

- **Novelty:** We developed a new webcam-captured Arabic numbers dataset to address the scarcity of numeric datasets. This dataset was collected from 19 participants using a simple and portable setup with a standard webcam, comprising a total of 950 eye-writing patterns.
- **Improved Preprocessing and Normalization Techniques:** We introduced a DFT-based normalization method that generates fixed-length representations while preserving spectral characteristics. Two types of data augmentation techniques and an initial-point normalization technique were applied to improve model robustness across various users and writing styles.
- **Hybrid Deep Learning Model:** The hybrid model integrates 1D CNN for local spatial feature extraction and TCN for modeling long-range temporal dependencies in the eye-writing patterns. This combination enhances recognition accuracy.
- **Robust Recognition and Empirical Validation:** To improve model robustness, we applied the proposed preprocessing techniques and data augmentation to mitigate artifacts commonly observed in real-world input data. In addition to accuracy, we evaluated precision, recall, and F1-score to provide a more comprehensive assessment of model performance. Extensive experiments show that the proposed system achieves high accuracy rates—97.68% on the webcam-captured dataset, 94.98% on the Japanese Katakana dataset, and 98.70% on the EOG-captured Arabic numbers dataset—under leave-one-subject-out (LOSO) cross-validation in user-independent settings, significantly outperforming existing state-of-the-art systems.

The layout of the remaining sections of this paper is outlined as follows: detailed descriptions of the datasets and methods employed in this research are presented in Section 2. In Section 3, we propose a novel framework for eye-writing recognition, which includes data augmentation, data preprocessing, and the architecture of the proposed model. In Section 4, we evaluate the performance of our proposed model using leave-one-subject-out (LOSO) cross-validation under user-independent settings. Lastly, we conclude this paper in Section 5 and discuss future work.

## 2. Dataset

We utilized two benchmark datasets and one newly created dataset for the evaluation of our proposed model. We present the details of these datasets in the following subsections, with particular focus on the methodology for collecting the newly created dataset.

### 2.1. EOG-Captured Arabic Numbers Dataset

**Figure 1:**
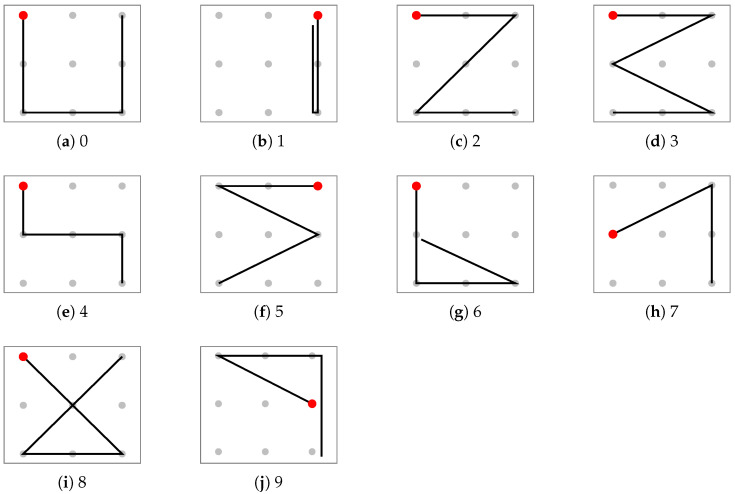
Eye-writing patterns for 10 Arabic numerals [5,8].

Chang et al. proposed 10 types of Arabic number patterns for eye-writing recognition [5], which are shown in Figure 1. Each pattern corresponds to Arabic numerals from 0 to 9. They collected EOG signals from 21 subjects, including three ALS patients and 18 healthy individuals, and the data from healthy subjects is publicly available. Within the healthy dataset, each pattern was recorded three times per subject, resulting in a total of 540 trials in the publicly available dataset.

### 2.2. Japanese Katakana Dataset

Fang and Shinozaki published an EOG dataset for Japanese Katakana characters [6]. They introduced 12 types of strokes, which can be combined to represent all characters in the Japanese Katakana. The dataset consists of isolated and continuous sequences, where continuous sequences are composed of multiple isolated sequences. The dataset was recorded from six healthy participants, and each subject performed 10 trials for each stroke. The procedure was designed in accordance with Lee et al. [4]. We used the isolated sequences for evaluation.

### 2.3. Proposed Webcam-Captured Arabic Numbers Dataset

This study introduced an original dataset of Arabic numbers captured via webcam. The dataset employs the same patterns proposed by Chang et al., as shown in Figure 1. We developed an eye-tracking system using a standard webcam, and the details of this system are described in the following section.

#### 2.3.1. Eye-Tracking System

**Figure 2:**
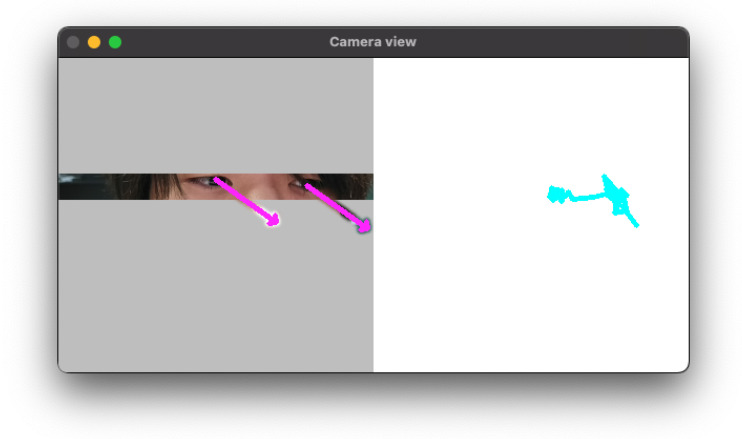
Interface of the eye-tracking system.

Our eye-tracking system captures eye positions and angles through a standard webcam. The system utilizes a publicly available gaze estimation model implemented with the OpenVINO framework [12]. The inference model processes three key inputs—left eye image, right eye image, and head angle to generate the corresponding three-dimensional gaze vectors. Complementary inference models automatically crop eye regions from the detected facial area. Figure 2 shows the application interface. The system calculates the eye-written point as the midpoint between the two gaze vectors, recording this two-dimensional coordinate for eye-writing recognition.

#### 2.3.2. Experimental Setup

**Figure 3:**
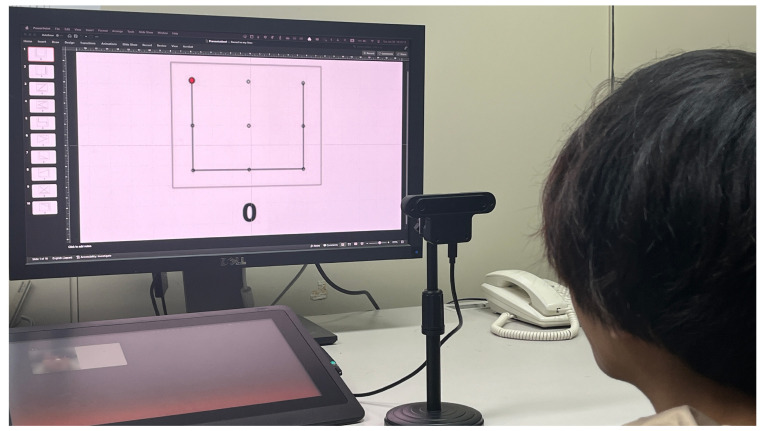
Setup of the experiment.

The setup of the experiment is shown in Figure 3. The subjects sat in front of a display, and a webcam was placed between the display and the subject. Eye-writing patterns were shown on the display, and the subjects moved their eyes along the patterns. The instructor guided the subjects and operated the system. In the experiment, a 24-inch display and a generic HD webcam were used.

#### 2.3.3. User Study

We conducted a user study to collect a dataset of eye-writing patterns using the developed eye-tracking system. The experiment consisted of two phases: practice and exercise.

In the practice phase, the users learned how to move their eyes with the instructor’s guidance. The users practiced moving their eyes along the patterns until they became familiar with the procedure. The patterns used in the practice phase were the same as those used in the exercise phase, and most users learned the procedure after practicing four patterns.

In the exercise phase, the users were asked to move their eyes along the patterns for each number. The users repeated the procedure for five rounds. After finishing the third round, the users took a five-minute break. The exercise phase took about 30 minutes, including the break.

**Figure 4:**
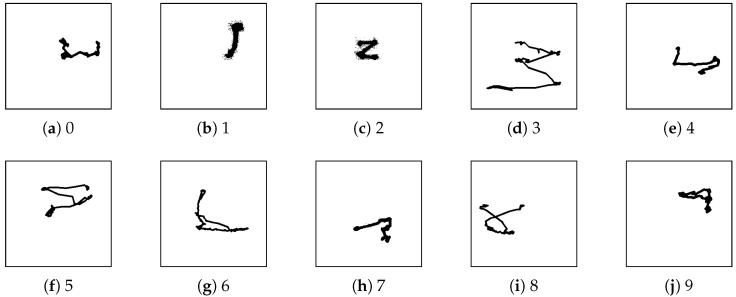
Examples of eye-writing patterns for webcam-captured Arabic numbers dataset.

The experiment was conducted with 19 users, and all users were healthy and had no experience with eye-writing. In total, 950 eye-writing patterns were collected. Figure 4 depicts examples of eye-writing patterns. The eye-writing positions and path characteristics varied between users.

## 3. Methods

We propose an advanced eye-writing recognition system to classify eye-writing patterns using deep learning techniques. The proposed system has three main components: data augmentation, preprocessing, and model architecture. The data augmentation component enhances the robustness of the model against variations such as stroke shape, speed, and timing. The preprocessing component normalizes the input data, keeping the essential features of the eye-writing patterns. The model component employs a hybrid architecture that combines 1D CNN and TCN to extract hierarchical features. The details of each component are described in the following subsections.

### 3.1. Data Augmentation

Data augmentation is a technique that increases sample size and variety, which is particularly important in scenarios where the available data is limited. This process helps prevent overfitting and improves the model’s generalization capabilities. In this work, we employed two data augmentation techniques: distortion-based augmentation and window warping.

#### 3.1.1. Data Augmentation with Distortion

**Figure 5:**
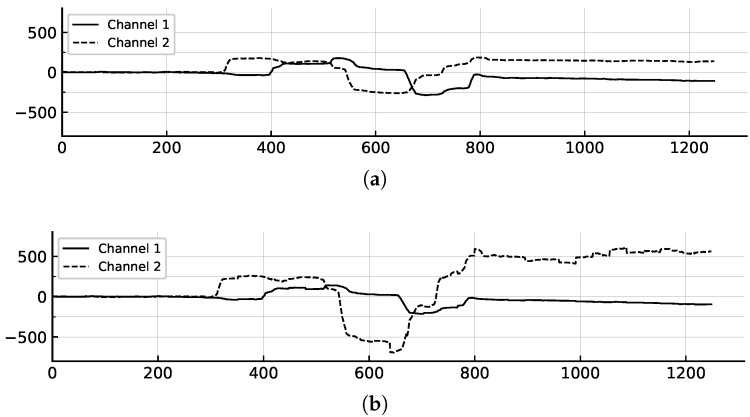
Original and distorted eye-writing patterns for “left, up, right, down, left” in Japanese Katakana dataset. (**a**) Original signal (**b**) Distorted signal with distortion applied. The normal distribution parameters are set to μ=1.01 and σ=0.1, and the gap *g* is set to 32.

To enhance the robustness of our model against variations in eye-writing patterns, we implemented a distortion-based data augmentation technique to generate additional training samples from the original training data. Inspired by [13], we introduce random scaling factors to the x and y coordinates of the eye movement data, simulating variations in writing speed and style. These scaling factors are sampled from a normal distribution, enabling controlled distortion while maintaining the overall structure of the eye-writing patterns as shown in Figure 5.

Let $X=[x_{0},x_{1},\ldots ,x_{N-1}]$ be a sequence of *N* points in two-dimensional space, where each point $x_{i}$ is represented as $x_{i}=\left[x_{i,0},x_{i,1}\right]$. The scaling factors $s_{x}$ and $s_{y}$ for the x and y coordinates are sampled from a normal distribution with mean μ and variance $\sigma^{2}$, that is,

(1) $$s_{x}∼N\left(\mu ,\sigma^{2}\right),\;s_{y}∼N\left(\mu ,\sigma^{2}\right)$$

These scaling factors are then applied to the sequence of points *X* as

(2) $$x_{j,0}\leftarrow x_{j,0}\cdot s_{x},\;x_{j,1}\leftarrow x_{j,1}\cdot s_{y}\;\forall j\geq i\;i\in \left\{0,\;g,\;2g,\ldots \right\},\;i+g<N$$

where *g* denotes the gap between points, and the transformation is applied at regular intervals starting from the initial point i=0.

The transformation process is applied to N/g segments of the sequence, beginning with the first point and proceeding sequentially through the sequence at intervals of *g* points. The transformed sequence $X^{\prime }$ serves as augmented training data. This augmentation strategy enhances the diversity of our training dataset, enabling the model to learn more robust features and achieve better generalization performance.

#### 3.1.2. Window Warping Data Augmentation

**Figure 6:**
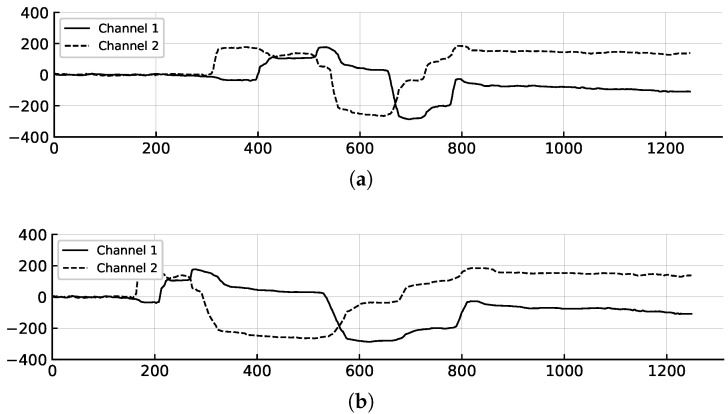
Original and window warped eye-writing patterns for the same pattern as in Figure 5. (**a**) Original signal. (**b**) Window warped signal with a portion of the time series data scaled by a factor of *s*, where the window size ratio *r* is set to 0.3 and the scaling factors *s* are set to 0.25 and 4.0.

In real-world scenarios, characteristics of the eye-writing strokes, such as speed and timing, can vary significantly among users. To address this variability, window warping data augmentation [14] partially warps the time axis of the eye-writing patterns. As shown in Figure 6, this technique involves selecting a portion of the time series data and scaling it to simulate different velocities and timings of strokes, which can help the model learn to recognize eye-writing patterns across time-varying characteristics.

Let *r* and *s* be the window size ratio and the scaling factor, respectively. During this process, a portion of time series data of size ⌈L·r⌉ is randomly selected. The selected portion is then interpolated to scale the time axis by a factor of *s*. Finally, the scaled data is concatenated with the remaining data, and the entire sequence is rescaled to maintain the original length *L*.

### 3.2. Data Preprocessing

In the preprocessing stage, robust handling of outlier inputs is essential for eye-writing recognition applications, especially in medical contexts. We implemented dataset-specific preprocessing techniques to enhance model accuracy by extracting key features while minimizing interference effects.

#### 3.2.1. Length Normalization with Discrete Fourier Transform

To standardize signal lengths, we utilize the Discrete Fourier Transform (DFT) processing [15]. Given a sequence x(n) with *L* samples, where n=0,1,…,L−1, its Fourier transform is

(3) $$X\left(k\right)=\sum_{n=0}^{L-1}x\left(n\right)e^{-j2\pi kn/L}\;k=0,1,\ldots ,L-1$$

For a target length of *M* samples where M<L, we truncate the frequency-domain representation to retain only the most essential spectral components and create $X_{d}\left(k\right)$,

(4) $$X_{d}\left(k\right)=X\left[\left(k+\left\lceil \frac{L-M}{2}\right\rceil \right)\;\mathrm{mod}\;L\right]\;k=0,1,\ldots ,M-1$$

Conversely, when M>L, we perform zero-padding in the frequency domain to extend the signal to the target length. Zero padding is applied symmetrically to both ends of the frequency spectrum to preserve the signal’s characteristics. The modified frequency-domain representation $X_{d}\left(k\right)$ is defined as

(5) $$X_{d}\left(k\right)=\left\{\begin{array}{cc} X[k] & \mathrm{for}\;0\leq k<\lceil L/2\rceil \\ 0 & \mathrm{for}\;\lceil L/2\rceil \leq k<M-\lfloor L/2\rfloor \\ X[k-(M-L)] & \mathrm{for}\;M-\lfloor L/2\rfloor \leq k<M \end{array}\right.$$

The fixed-length sequence $x_{d}\left(n\right)$ is then derived by

(6) $$x_{d}\left(n\right)=\frac{1}{M}\sum_{k=0}^{M-1}X_{d}\left(k\right)e^{j2\pi kn/M}\;n=0,1,\ldots ,M-1$$

**Figure 7:**
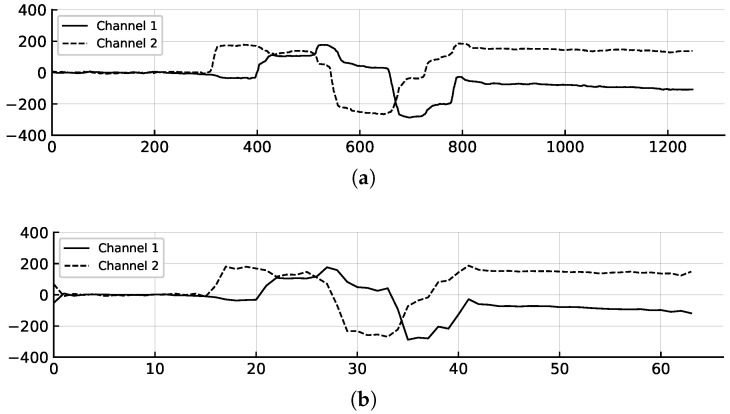
Original and normalized eye-writing patterns. (**a**) Original signal with 1250 samples. (**b**) Length-normalized signal with DFT-based normalization applied, resulting in a sequence of 64 samples.

This approach produces a fixed-length sequence $x_{d}\left(n\right)$ with *M* samples that is suitable for batch processing in deep learning models, as shown in Figure 7. By removing the high-frequency components using DFT-based normalization, it effectively reduces noise and outliers while preserving the essential spectral characteristics of the original signal.

#### 3.2.2. Initial-Point Normalization

Eye-writing patterns exhibit positional variability across trials and users, complicating direct comparison. The initial-point normalization technique [16] addresses this challenge by aligning each pattern to a common reference point. For an eye-writing pattern $P=[p_{0},p_{1},\ldots ,p_{N-1}]$ with *N* points, where each point $p_{i}=\left[x_{i},y_{i}\right]$, the normalization is defined as:

(7) $$p_{i}^{\prime }=\left[x_{i}-x_{0},y_{i}-y_{0}\right]\;\forall i\in \left\{0,1,\ldots ,N-1\right\}$$

where $p_{i}^{\prime }$ represents the normalized point at index *i*.

This transformation shifts the entire sequence to make the initial point $p_{0}$ the origin (0,0). By compensating for positional variations, this normalization significantly improves model robustness, enabling more effective pattern recognition across different writing instances.

### 3.3. Model Architecture

**Figure 8:**
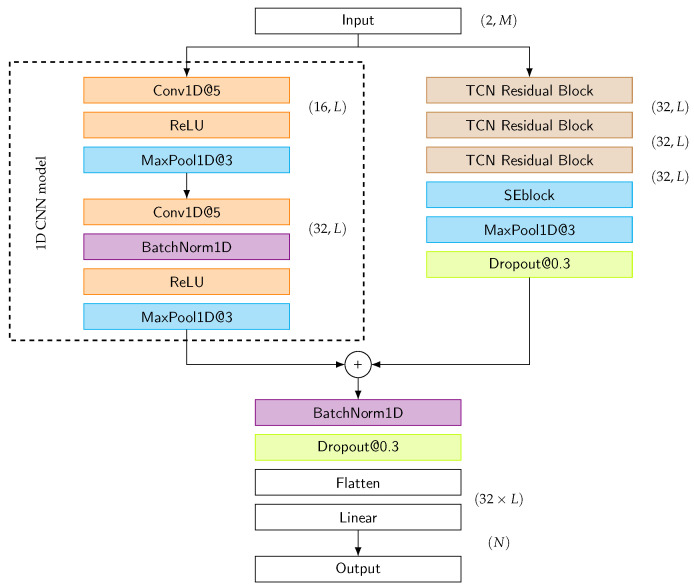
The architecture of the 1D CNN-TCN model.

Figure 8 depicts the proposed model, which takes an input of dimension L×2, where *L* represents the length of the signal and 2 represents the x and y coordinates of eye movement. The architecture consists of two parallel branches: a 1D CNN branch and a TCN branch. The choice of *L* directly affects the parameter size and computational cost of the model. In real-world applications, inference time and model compactness are crucial considerations. Therefore, simpler and more efficient models with smaller *L* are preferred, provided they maintain acceptable accuracy. This hybrid architecture effectively leverages both the spatial feature extraction capabilities of CNNs and the temporal modeling strengths of TCNs. This design makes it well-suited for eye-writing recognition where both spatial patterns and temporal dynamics are important.

In the 1D CNN branch, the first convolutional layer applies filters of size 5 to produce 16 feature maps with an appropriate padding setting to maintain input sequence length. This is followed by a MaxPool layer with a kernel size of 3, with stride and padding of 1, to reduce temporal resolution. The second convolutional layer takes these 16 channels and outputs 32 feature channels using the same filter size, with subsequent batch normalization and ReLU activation for non-linearity, followed by another MaxPool operation for further dimensionality reduction.

Meanwhile, the TCN branch processes the same input using a kernel size of 3 with three layers of 32 filters each, incorporating a dropout rate of 0.3 for regularization and a Squeeze-and-Excitation block to emphasize important features. The outputs from both branches are then combined and processed through batch normalization and dropout for additional regularization. Finally, regularized values are flattened to a multi-dimensional vector, which is mapped to corresponding classes via a linear layer.

#### 3.3.1. Temporal Convolutional Block

**Figure 9:**
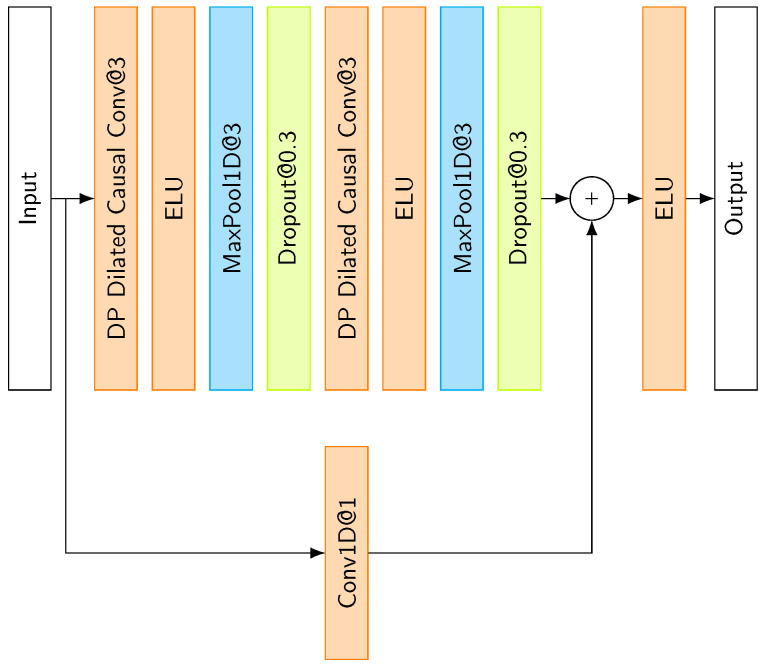
Temporal Convolutional Block architecture.

Our model employs a TCN block as illustrated in Figure 9. Unlike the original TCN block, we implement a depthwise–pointwise convolutional layer in place of a standard convolutional layer. The detailed architecture of the depthwise–pointwise convolutional layer is described in the following section. Following [17], we incorporate a batch normalization layer [18] and ELU activation function. Additionally, we utilize a max pooling layer to downsample the feature maps following the second activation function.

#### 3.3.2. Depthwise–Pointwise Dilated Causal Convolutional Layer

**Figure 10:**
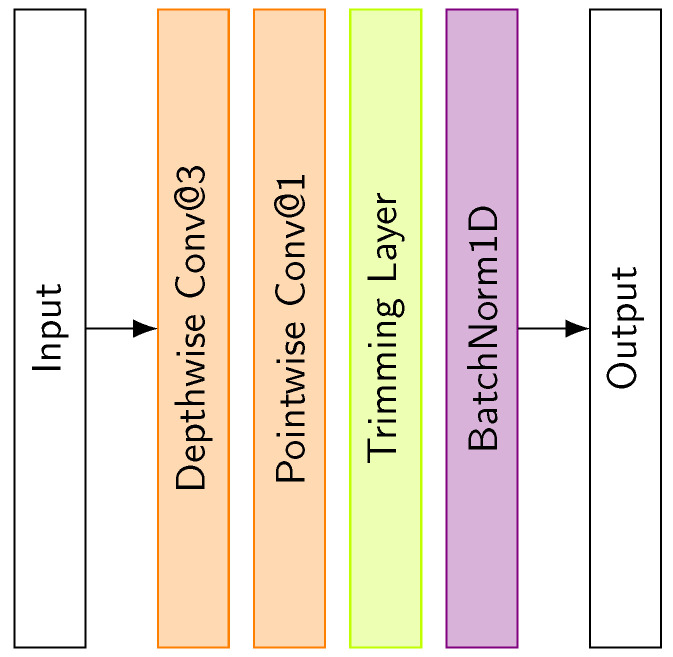
Architecture of the Depthwise–Pointwise Dilated Causal Convolutional Layer.

The proposed TCN block incorporates a depthwise–pointwise dilated causal convolution as an alternative to a standard convolutional layer. Figure 10 showcases the architecture of this layer. In this architecture, the depthwise convolutional layer [19] applies a filter of specified size to each input channel independently. This is followed by a pointwise convolutional layer that applies a 1×1 convolution to combine the outputs. This architectural choice significantly reduces both the number of parameters and computational complexity while preserving the network’s capability to learn complex temporal patterns.

#### 3.3.3. Squeeze-And-Excitation Block

The Squeeze-and-Excitation (SE) block [20] is a lightweight attention mechanism that dynamically recalibrates channel-wise feature maps by modeling interdependencies between channels. The SE block consists of two main operations: squeeze and excitation. The squeeze operation aggregates global spatial information through global average pooling to generate channel-wise statistics as a descriptor. The excitation operation applies a fully connected layer to these statistics, constructing weights that represent the importance of each channel. These weights are then used to scale the original feature maps. This mechanism enables the model to adaptively emphasize informative features while suppressing less relevant ones, thereby enhancing the overall recognition performance.

## 4. Results and Discussion

We conducted evaluations on three datasets introduced in Section 2. To this end, we designed an emprical evaluation protocol, which is described in the first part of this section. After that, we present the results of our proposed model and compare them with existing state-of-the-art results. Finally, we evaluate each component of our proposed preprocessing, augmentation, and model architecture to demonstrate the effectiveness of our approach.

### 4.1. Evaluation Protocol

We evaluated our model using leave-one-subject-out (LOSO) cross-validation under user-independent conditions. In this approach, the evaluation process iterates through each subject in the dataset. Data from all subjects except one is used for training, while the remaining subject’s data is used exclusively for testing. This methodology ensures the model is evaluated on completely unseen subjects, providing a rigorous assessment of its generalization capabilities across different individuals.

**Figure 11:**
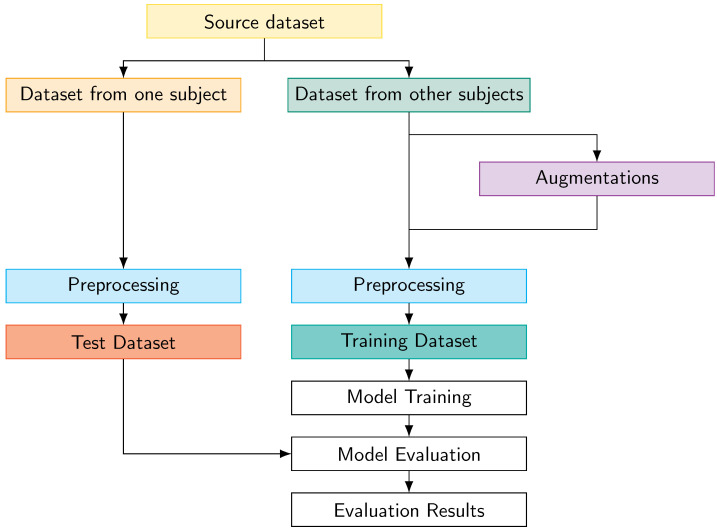
Block diagram of the proposed evaluation protocol showing the data flow from source dataset through augmentation, preprocessing, training, and evaluation stages.

Figure 11 illustrates our evaluation framework. The protocol begins by splitting the source dataset into two separate portions: one containing data from a single subject (for testing) and another comprising data from all other subjects (for training). The training data undergoes data augmentation. We implemented three augmentation techniques, which are described in the next subsection. Both the augmented and original training data are preprocessed before model training. Similarly, the test data undergoes preprocessing before evaluation. This systematic approach ensures an unbiased assessment while maintaining strict separation between training and testing data, which is crucial for validating the model’s ability to generalize to new users.

**Figure 12:**
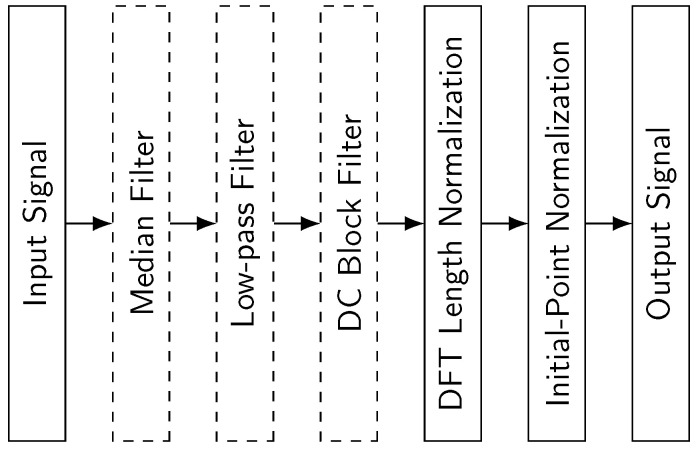
Sequential preprocessing pipeline for EOG signal processing. The pipeline shows the six processing stages applied to enhance signal quality and prepare data for the recognition model. The three filters represented by dashed boxes are not applied to the webcam-captured Arabic numbers dataset.

In preprocessing, we implemented a pipeline of steps illustrated in Figure 12. Raw eye-writing sequences often contain noise and unwanted artifacts that reduce recognition accuracy. Following the approach of [6], we applied a sequence of filters to the raw eye-writing data from two EOG-captured datasets. First, the input signal was processed with a median filter to remove sporadic noise. Next, a low-pass filter was applied to eliminate high-frequency components irrelevant to recognition. Then, the signal was passed through a direct current (DC) block filter that removes any constant offset in the signal, centering the values around zero. After these filtering steps, the Discrete Fourier Transform (DFT) length normalization was applied to standardize the signal length, followed by initial-point normalization to align the initial point of each eye-writing pattern to the origin. For the webcam-captured Arabic numbers dataset, the first three filters (dashed boxes in Figure 12) were not applied due to the different nature of data capture.

### 4.2. Experimental Settings

The deep learning models were implemented using the PyTorch framework in Python 3.10. Experiments were conducted on a desktop computer equipped with an Intel Core i5-10400 CPU (Intel, Santa Clara, CA, USA), NVIDIA GeForce RTX 3060 GPU (CUDA 12.4) (NVIDIA, Santa Clara, CA, USA), and 48 GB RAM.

We employed the Adam optimizer with AMSGrad for model training and set the L2 weight decay to 0.1 to mitigate overfitting. The cross-entropy loss function was used to measure the discrepancy between predicted and true labels. The cross-entropy loss is defined as

(8) $$L=-\frac{1}{N}\sum_{i=1}^{N}\sum_{j=1}^{C}y_{ij}\log\left({\hat{y}}_{ij}\right)$$

where *N* is the number of samples, *C* is the number of classes, $y_{ij}$ is a binary indicator (0 or 1) if class label *j* is the correct classification for sample *i*, and ${\hat{y}}_{ij}$ is the predicted probability that sample *i* belongs to class *j*. In all experiments, the model was trained for 400 epochs with a batch size of 512. We evaluated performance metrics such as accuracy, precision, recall, and F1-score after training.

Hyperparameters such as the batch size, number of epochs, weight decay, and number of cosine annealing iterations were determined through grid search and empirical experiments to optimize performance across all datasets.

Common hyperparameter settings for all datasets are detailed in Table 2. Regarding two types of distortion data augmentation, the normal distribution parameters are different, where the type I distortion has a mean μ=1.0 and variance $\sigma^{2}=0.1$, while the type II distortion has a mean μ=1.01 and variance $\sigma^{2}=0.01$. The gap *g* for both types of distortion is dataset-specific. The learning rate was dynamically adjusted during the training process, initialized at 0.001 and regulated through a cosine annealing [21] scheduler. The period of the cosine annealing was set to 16 iterations.

In the filtering process for the Japanese Katakana and EOG-captured Arabic datasets, dataset-specific cutoff frequencies were established based on each dataset’s sampling rate, as presented in Table 3. The gap *g* for the two types of distortion was set to 64 for the EOG-captured Arabic dataset and 32 for other datasets.

### 4.3. Performance Metrics

For evaluation, we use standard performance metrics including accuracy, precision, recall, and F1-score.

Furthermore, hypothesis testing is conducted to statistically analyze whether the proposed approach is significantly better than the baseline method. To calculate the *p*-value, we employ the two-tailed Wilcoxon signed-rank test [22] with a significance level of 0.05. A *p*-value less than the significance level indicates that the null hypothesis can be rejected, suggesting that the observed difference is unlikely to be due to chance.

### 4.4. Results in Japanese Katakana Dataset and Comparison with State-of-the-Art

Table 4 presents the performance metrics of the proposed 1D CNN-TCN model across different subjects in the Japanese katakana dataset. The proposed model achieved an average accuracy of 94.48% with a standard deviation of 5.05%. The fourth, fifth, and sixth subjects exhibited lower accuracy primarily due to the presence of signal outliers in their EOG recordings. While the model performed the lowest on the fourth subject at 85.12%, it still achieved higher accuracy than 90% for all subjects except the fourth subject.

We summarize previous works in Table 5 to compare the performance of the proposed model with other methods, including various evaluation strategies such as user-independent and user-dependent evaluations. Our model outperformed existing methods, achieving the highest accuracy of 94.48% under an unbiased user-independent evaluation strategy. The integration of our 1D CNN-TCN architecture with the proposed preprocessing and data augmentation techniques has proven effective for eye-writing stroke recognition, particularly in challenging user-independent scenarios.

### 4.5. Results in EOG-Captured Arabic Numbers Dataset and Comparison with State-of-the-Art

In the EOG-captured Arabic numbers dataset, the proposed 1D CNN-TCN model achieved an average accuracy of 98.70% with a standard deviation of 2.26% across 18 subjects, as shown in Table 6. All subjects achieved an accuracy equal to or greater than 90%, with the majority of subjects achieving 100% accuracy.

Table 7 compares the performance of the proposed model with other methods, which demonstrates that the proposed model outperforms existing methods in terms of accuracy under consistent user-independent evaluations. The results indicate that the proposed model effectively captures the unique characteristics of eye-writing strokes, leading to high recognition accuracy across different subjects.

### 4.6. Results in Webcam-Captured Arabic Numbers Dataset

In the webcam-captured Arabic numbers dataset, the proposed model achieved an average accuracy of 97.68% with a standard deviation of 3.63% across 19 subjects, as shown in Table 8. Of the subjects, 10 out of 19 achieved 100% accuracy, while the remaining subjects achieved accuracies ranging from 88.00% to 98.00%. Our model demonstrates effectiveness and robustness in recognizing eye-writing strokes captured through webcams rather than EOG sensors. This performance indicates that the model can perform well in a wider range of scenarios, which is crucial for practical applications in real-world situations.

### 4.7. Ablation Experiments

We conducted ablation experiments to evaluate the individual contribution of each component in our proposed model. Specifically, we systematically evaluated the model by adding or removing three key components: DFT length normalization, initial-point normalization, and data augmentation techniques.

The baseline 1D CNN-TCN model was compared against various configurations to isolate the impact of each component and their combinations. Since eye-writing speed, duration, and motion range vary considerably across subjects and datasets, we standardized input lengths in the baseline model by zero-padding the raw signals to fixed lengths specific to each dataset. Although zero-padding may introduce some noise, to minimize its impact, we applied zero-padding to the raw input signals before feature extraction, ensuring it occurred prior to the fully connected layer. In the baseline model, raw signals were zero-padded to fixed lengths of 1300, 1800, and 512 samples for the Japanese Katakana, EOG-captured Arabic numbers, and webcam-captured Arabic numbers datasets, respectively, without truncation. This naive approach serves as a reference point to assess the effectiveness and fairness of our proposed preprocessing and augmentation techniques.

Table 9 presents the results of these experiments across different datasets. The configurations tested include: (1) the baseline 1D CNN-TCN model without any preprocessing or augmentation, (2) the model with DFT length normalization, (3) the model with DFT length normalization and initial-point normalization, (4) the model with DFT length normalization and data augmentations, and (5) the full model with DFT length normalization, initial-point normalization, and data augmentations. In all configurations, the filtering steps (median filter, low-pass filter, and DC block filter) were consistently applied to the Japanese Katakana and EOG-captured Arabic numbers datasets, as these filters are essential for enhancing signal quality. The webcam-captured Arabic numbers dataset did not include these filtering steps, as the raw signals were already of sufficient quality for effective processing by the model. Each measurement represents the average across LOSO cross-validation for all subjects in corresponding datasets.

Analysis of the results reveals several findings across all datasets. The partial model with DFT length normalization achieved outstanding improvements in accuracy compared to the baseline model: 15.99 pp for the Japanese Katakana dataset (87.74% vs. 71.75%), 27.97 pp for the EOG-captured Arabic numbers dataset (96.30% vs. 68.33%), and 34.42 pp for the webcam-captured Arabic numbers dataset (95.37% vs. 60.95%). This improvement is attributed to the effective normalization technique, which enhances feature extraction capabilities.

In addition, initial-point normalization and data augmentations also contributed to performance improvements complementary to DFT length normalization. The full model improved the accuracy by 2.76 and 4.94 pp for the Japanese Katakana dataset compared to models without initial-point normalization and without augmentations, respectively. For the EOG-captured Arabic numbers dataset, the full model improved the accuracy by 2.22 and 1.29 pp compared to models without initial-point normalization and without augmentations, respectively. For the webcam-captured Arabic numbers dataset, the full model improved the accuracy by 2.31 and 0.73 pp compared to models without initial-point normalization and without augmentations, respectively. These results indicate that both initial-point normalization and data augmentations effectively enhance the model’s robustness against a wider variety of eye-writing patterns.

Table 10 shows the calculated *p*-values for the ablation study comparing the baseline model with each of the other configurations across all datasets. Consequently, all *p*-values were found to be less than the significance level of 0.05, indicating that improvements in accuracy were statistically significant relative to the baseline across all configurations.

Table 11 presents the results of the ablation experiments on the fusion components. By comparing the performance of the 1D CNN, TCN, and their combination in both parallel and serial integrations (where 1D CNN output is fed to TCN), it is evident that the parallel model outperforms both individual models and the serial fused model. Across all datasets, the parallel model achieved the highest accuracy, demonstrating the effectiveness of the proposed model and the benefits of the fusion architecture.

## 5. Conclusions

This paper introduces a novel eye-writing system leveraging vision-based eye-tracking technologies, offering a practical solution for individuals with muscle control difficulties. The proposed system effectively classifies corresponding eye-writing patterns with high accuracy 97.68% for webcam-captured Arabic numbers, 94.98% for Japanese Katakana, and 98.70% for EOG-captured Arabic numbers) using a hybrid approach that integrates 1D-CNN and Temporal Convolutional Networks (TCN). Our experiments demonstrate the potential of using a standard webcam for gaze tracking, eliminating the need for specialized equipment like electrooculograms (EOG) or infrared cameras. The webcam-based approach not only simplifies the setup but also enhances accessibility, making eye-writing a more viable and user-friendly interface for assistive technologies. While our system shows promising results, future work should focus on real-time implementation and evaluation with a larger, more diverse population of users with various mobility impairments.

## Figures and Tables

**Table 1:** Existing eye-writing recognition systems.

Authors	Method	Characters	Metric Score (%)
Tsai et al. [3]	Heuristic algorithm	10 numerals and 4 symbols	75.5
Lee et al. [4]	DTW	26 alphabets and 3 symbols	87.38
Chang et al. [5]	DPW, SVM	10 Arabic numbers	95.74
Fang & Shinozaki [6]	HMM, DTW	12 Japanese Katakana	77.6 (DTW) 86.5 (HMM)
Chang et al. [8]	DNN	10 Arabic numbers	97.78
Zou & Zhang [7]	CNN	Japanese Katakana	92.64
Dai et al. [9]	1D CNN-LSTM	Japanese Katakana	95 (Word) 97 (Character)
Bature et al. [10]	TCN	10 Arabic numbers	93.51

**Table 2:** Hyperparameter settings used in the experiments.

Parameter Settings			
Epochs	400	Length of preprocessed input	64
Batch size	512	Cosine annealing iterations	16
Weight decay	0.1	Maximum learning rate	0.001
AMSGrad	True	Minimum learning rate	dataset-specific
**Data Augmentation with Distortion I**			
Mean μ		1.0	
Variance $\sigma^{2}$		0.1	
Gap *g*		dataset-specific	
**Data Augmentation with Distortion II**			
Mean μ		1.01	
Variance $\sigma^{2}$		0.01	
Gap *g*		dataset-specific	
**Window Warping Data Augmentation**			
Window size ratio *r*		0.3	
Scaling factor(s) *s*		0.25 and 4.0	

**Table 3:** Dataset-specific settings for preprocessing.

Settings	Japanese Katakana	EOG-Captured Arabic	Webcam-Captured Arabic
Cosine annealing iterations	16	16	16
Minimum learning rate	0.0	$1.0\times 10^{-6}$	0.0
Filter order	5	5	None
Sampling rate (Hz)	125	64	None
Cutoff frequency (Hz)	10	10	None
Gap *g* for distortion type I	32	64	32
Gap *g* for distortion type II	32	64	32

**Table 4:** Performance metrics across different subjects in Japanese Katakana dataset.

Subject	Loss	Accuracy (%)	Precision (%)	Recall (%)	F1-Score (%)
Subject-1	0.0838	99.17	99.24	99.17	99.20
Subject-2	0.0516	100.00	100.00	100.00	100.00
Subject-3	0.2381	96.67	97.22	96.89	97.06
Subject-4	0.6156	85.12	87.63	85.00	86.30
Subject-5	0.3404	91.67	94.08	91.67	92.86
Subject-6	0.4187	94.26	94.71	94.24	94.47
Average	0.2914	94.48	95.48	94.49	94.98
Std. Dev.	0.1946	5.05	4.12	5.10	4.61

**Table 5:** Comparison for the Japanese katakana dataset for eye-writing stroke recognition.

Author	Method	Evaluation Strategy	Accuracy (%)
Fang & Shinozaki [6]	DTW	User independent	77.60
	HMM		86.50
	GMM-HMM	User dependent	93.50
	DNN-HMM		93.80
Zou & Zhang [7]	CNN-stroke	Leave-one-trial-out	92.64
Bature et al. [10]	TCN	Train–test split (70:30)	93.51
Suzuki et al. (This work)	1D CNN-TCN	User independent (LOSO)	94.48

**Table 6:** Performance metrics across different subjects in EOG-captured Arabic numbers dataset.

Subject	Loss	Accuracy (%)	Precision (%)	Recall (%)	F1-Score (%)
Subject-1	0.1496	100.00	100.00	100.00	100.00
Subject-2	0.2315	100.00	100.00	100.00	100.00
Subject-3	0.2358	96.67	97.50	96.67	97.08
Subject-4	0.0250	100.00	100.00	100.00	100.00
Subject-5	0.3074	93.33	95.00	93.33	94.16
Subject-6	0.1964	93.33	95.00	93.33	94.16
Subject-7	0.1045	100.00	100.00	100.00	100.00
Subject-8	0.0256	100.00	100.00	100.00	100.00
Subject-9	0.0152	100.00	100.00	100.00	100.00
Subject-10	0.1361	96.67	97.50	96.67	97.08
Subject-11	0.0871	100.00	100.00	100.00	100.00
Subject-12	0.0260	100.00	100.00	100.00	100.00
Subject-13	0.1022	100.00	100.00	100.00	100.00
Subject-14	0.1059	100.00	100.00	100.00	100.00
Subject-15	0.1538	100.00	100.00	100.00	100.00
Subject-16	0.0743	100.00	100.00	100.00	100.00
Subject-17	0.2079	96.67	97.50	96.67	97.08
Subject-18	0.1328	100.00	100.00	100.00	100.00
Average	0.1287	98.70	99.03	98.70	98.86
Std. Dev.	0.0807	2.26	1.70	2.26	1.98

**Table 7:** Comparison for the EOG-captured Arabic numbers dataset for eye-writing stroke recognition.

Author	Method	Evaluation Strategy	Accuracy (%)
Chang et al. [5]	DTW	User independent	92.41
	DPW		94.07
	DTW-SVM		94.08
	DPW-SVM		95.74
Chang et al. [8]	DNN		97.78
Suzuki et al. (This work)	1D CNN-TCN	User independent (LOSO)	98.70

**Table 8:** Performance metrics across different subjects in the webcam-captured Arabic numbers dataset.

Subject	Loss	Accuracy (%)	Precision (%)	Recall (%)	F1-Score (%)
Subject-1	0.5338	88.00	89.17	88.00	88.58
Subject-2	0.1075	98.00	98.33	98.00	98.17
Subject-3	0.1061	100.00	100.00	100.00	100.00
Subject-4	0.0580	100.00	100.00	100.00	100.00
Subject-5	0.2049	98.00	98.33	98.00	98.17
Subject-6	0.1078	100.00	100.00	100.00	100.00
Subject-7	0.2189	96.00	96.67	96.00	96.33
Subject-8	0.0718	100.00	100.00	100.00	100.00
Subject-9	0.2372	96.00	96.67	96.00	96.33
Subject-10	0.1714	98.00	98.33	98.00	98.17
Subject-11	0.0688	100.00	100.00	100.00	100.00
Subject-12	0.2907	96.00	96.67	96.00	96.33
Subject-13	0.0378	100.00	100.00	100.00	100.00
Subject-14	0.0307	100.00	100.00	100.00	100.00
Subject-15	0.1593	100.00	100.00	100.00	100.00
Subject-16	0.0464	98.00	98.33	98.00	98.17
Subject-17	0.0542	100.00	100.00	100.00	100.00
Subject-18	0.0676	100.00	100.00	100.00	100.00
Subject-19	0.4437	88.00	91.25	88.00	89.60
Average	0.1588	97.68	98.09	97.68	97.89
Std. Dev.	0.1353	3.63	2.98	3.63	3.30

**Table 9:** Ablation study showing the impact of different components on model accuracy.

Model	Accuracy (%)		
	Japanese Katakana	EOG-Captured Arabic	Webcam-Captured Arabic
1D CNN-TCN (baseline)	71.75	68.33	60.95
+ DFT Length Normalization	87.74	96.30	95.37
+ DFT LN + Initial-Point Normalization	89.54	97.41	96.95
+ DFT LN + Augmentations	91.72	96.48	95.37
+ DFT LN + IP Norm + Augs	94.48	98.70	97.68

**Table 10:** *p*-value analysis for ablation study.

Model	*p*-Value		
	Japanese Katakana	EOG-Captured Arabic	Webcam-Captured Arabic
1D CNN-TCN (baseline)	-	-	-
+ DFT Length Normalization	0.031250	0.0001954	0.0001312
+ DFT LN + Initial-Point Normalization	0.031250	0.0001954	0.0001312
+ DFT LN + Augmentations	0.031250	0.0001944	0.0000038
+ DFT LN + IP Norm + Augs	0.031250	0.0001944	0.0001312

**Table 11:** Ablation study showing the impact of different fusion components on model accuracy.

Model	Accuracy (%)		
	Japanese Katakana	EOG-Captured Arabic	Webcam-Captured Arabic
1D CNN	93.21	98.33	97.58
TCN	85.49	96.44	92.32
1D CNN-TCN (Serial)	91.43	97.22	96.74
1D CNN-TCN (Parallel)	94.48	98.70	97.68

## Data Availability

The data supporting the findings of this study are available at https://u-aizu.ac.jp/labs/is-pp/pplab/eyewriting/eye-writing_data.zip (accessed on 3 October 2025).
